# Improved ground truth annotation by multimodal image registration from 3D ultrasound to histopathology for resected tongue carcinoma

**DOI:** 10.1007/s00405-024-08979-1

**Published:** 2024-09-30

**Authors:** N. M. Bekedam, M. J. A. van Alphen, E. M. V. de Cuba, L. H. E. Karssemakers, M. B. Karakullukcu, L. E. Smeele

**Affiliations:** 1 Department of Head and Neck Surgery and Oncology, Netherlands Cancer Institute, Antoni Van Leeuwenhoek, Plesmanlaan 121, 1066 CX Amsterdam, The Netherlands; 2 Academic Centre of Dentistry Amsterdam, Vrije Universiteit, Gustav Mahlerlaan 3004, 1081 LA Amsterdam, The Netherlands; 3 Department of Head and Neck Surgery and Oncology, Verwelius 3D Lab, Netherlands Cancer Institute, Antoni Van Leeuwenhoek, Amsterdam, The Netherlands; 4 Department of Pathology, Netherlands Cancer Institute, Antoni Van Leeuwenhoek, Amsterdam, The Netherlands

**Keywords:** 3D ultrasound, Annotation, Deep learning, Pathology, Registration, Resection margin

## Abstract

**Objectives:**

This study’s objectives are (1) to investigate the registration accuracy from intraoperative ultrasound (US) to histopathological images, (2) to assess the agreement and correlation between measurements in registered 3D US and histopathology, and (3) to train a nnUNet model for automatic segmentation of 3D US volumes of resected tongue specimens.

**Methods:**

Ten 3D US volumes were acquired, including the corresponding digitalized histopathological images (n = 29). Based on corresponding landmarks, the registrations between 3D US and histopathology images were calculated and evaluated using the target registration error (TRE). Tumor thickness and resection margins were measured based on three annotations: (1) manual histopathological tumor annotation (HTA), manual 3D US tumor annotation, and (2) the HTA registered in the 3D US. The agreement and correlation were computed between the measurements based on the HTA and those based on the manual US and registered HTA in US. A deep-learning model with nnUNet was trained on 151 3D US volumes. Segmentation metrics quantified the model’s performance.

**Results:**

The median TRE was 0.42 mm. The smallest mean difference was between registered HTA in US and histopathology with 2.16 mm (95% CI − 1.31; 5.63) and a correlation of 0.924 (p < 0.001). The nnUNet predicted the tumor with a Dice similarity coefficient of 0.621, an average surface distance of 1.15 mm, and a Hausdorff distance of 3.70 mm.

**Conclusion:**

Multimodal image registration enabled the HTA’s registration in the US images and improved the agreement and correlation between the modalities. In the future, this could be used to annotate ground truth labels accurately.

## Introduction

The high frequency of involved or close resection margins in oncological surgery of the tongue necessitates the implementation of an intraoperative margin assessment tool, such as ultrasound (US) [1]. Based on the tumor annotation in US, resection margins in a resected specimen can be assessed [2]. Medical image segmentation tasks are often performed automatically by deep learning models. These models are less time-consuming and reduce the operator variability [3, 4]. Training a deep learning model for automatic segmentation requires a large amount of data and accurate annotation of these data. Data consist of images with corresponding ground truth labels, which are segmentation maps delineating the target areas. These ground truth labels are often created manually, which is considered the most accurate segmentation. Incorrectly annotated ground truth labels could negatively influence the final performance of a deep learning model [4]. The quality of the ground truth labels is influenced by multiple factors, such as the annotator’s experience, interobserver variability, and the distinguishability of the target area within the imaging modality. Multimodality image registration could help annotate the target area in one modality and map this annotation to the other modality [4–6].

For tongue carcinomas, the initial deep-learning model has been advanced to a model that automatically segments the tumor and specimen regions in 3D US volumes [7, 8]. This model improved the intra-operative segmentation time significantly, but the correct analysis of the final margin status of the resected specimen did not correlate to histopathology (r = 0.12; p = 0.67) [8]. Reasons for this inaccuracy were the manual tumor annotation, the limited spatial resolution of US, the unknown measurement location in histopathology, and the tissue shrinkage when handling in the lab.

Another study shows that the agreement of 3D US compared to histopathology was 2.3 mm (SD ± 3.34) with a correlation of 0.73 [9]. A more objective method to compare the margin assessed in US and histopathology was used to tackle the problem of unknown measurement locations in histopathology. The lower and upper boundaries of the agreement still showed inaccuracies. Due to deformations, an irregular shape, and heterogeneous characteristics of resected tissue, tongue carcinoma in 3D US volumes was difficult to annotate manually. Histopathology is the gold standard in diagnostics due to its high-resolution whole-slide images (WSI). Consequently, manual histopathological tumor annotation (HTA) is the highest quality. Multimodal image registration from the US images to the corresponding WSI could enable the utilization of the HTA in the US images. With the HTA as an overlay in the US image, high-quality ground truth labels of the US images could be created for deep learning training.

The goal of this study is to create ground truth annotation of US images using histopathology tumor annotation. The objectives are (1) to investigate the registration accuracy of 3D US to postoperative histopathology slides, (2) to assess the agreement and correlation between the resection margin and tumor thickness in histopathology and US with registered annotation, and (3) to train a new version of the deep learning model for automatic segmentation of ex-vivo tongue carcinoma in 3D US volumes. It is hypothesized that registered HTA in US images provides more agreement and correlation than the manual annotation in US images between the measurements in US and histopathology. This would substantiate that registered HTA in US images functions as a more reliable ground truth label for deep learning models than manual annotation of US images.

## Method

### Study population and materials

This study included ten patients with squamous cell carcinoma of the tongue. This study was approved by the medical ethics committee (NL-78804.031.21). All patients included signed an informed consent form before enrollment. During surgery, a 3D US volume of the specimen was acquired using a 10 MHz transducer (I14C5T, BK Medical, Denmark) with a BK5000 US system (software version: 5.148.18234.26, license: OEM interface, BK Medical, Denmark) attached to a motorized system (Master 2s, NEJE, China). The 3D US acquisition is described in an earlier article [7]. Pathological whole slide images (WSI) were obtained with a digital scanner (software version 3.2.1, Pannoramic 1000, 3DHistech, Budapest, Hungary) and exported with SlideViewer (version 2.6, 3DHistech Ltd., Budapest, Hungary). Annotations, transformations, and registrations were performed using 3DSlicer (version 5.6.1), a free open-source software application for medical image computing [10]. Statistical analysis was performed in SPSS (version 29.0, IBM, Chicago, IL, USA) [11]. The deep learning models were set up with the nnUNet version 2 (nnUNetv2) platform [12]. Training and evaluation were performed on a workstation with Windows subsystem for Linux (Ubuntu 22.04; 2 × NVIDIA GeForce RTX2080 11264MiB GPUs; Intel Xeon Gold 6130 2.10 GHz CPU).

### Data acquisition

After resecting the carcinoma, up to three 16-gauge intravenous cannulas were inserted as fiducial markers in the specimen’s longest dimension. The 3D US volumes were acquired and reconstructed with 0.5 mm slice spacing between the US images.

At the Department of Pathology, the specimen, including the cannulas, was fixated and inked for proper orientation. Prior to slicing, the cannulas were removed, leaving artificial round defects in the tissue. The specimen was then cut into four mm thick slides parallel to the US images for paraffin embedding. Slides not fitting into the cassettes were subdivided into smaller parts. Four μm slices were cut from the paraffin slides, mounted on glass, stained with hematoxylin and eosin (H&E), and digitally scanned for histopathological analysis. The WSI without tumor and subdivided slides were excluded for the registration analysis and the assessment of agreement and correlation between histopathology and US with HTA. The number of included slides is reported in the Results, Registration section.

### Dataset nnUNetv2

A different dataset was used to train the nnUNet model in this study. Our first nnUNet model was developed with the nnUNetv1 platform [8]. This model was trained on the old dataset of 89 3D US volumes of 22 patients, of which a technical physician manually annotated the ground truth labels. In the current study, we used the nnUNetv2 platform, and the old dataset was extended to 151 volumes from 52 patients. This extension also included the ten patients for the registration analysis and agreement assessment. All 3D US volumes and corresponding ground truth labels were stored in the Medical Segmentation Decathlon format. Ground truth labels, which a technical physician manually annotated, consisted of background (region = 0), specimen (region = 1), and tumor (region = 2). Except for the included WSI with registered HTA in US, the registered HTA was used as a ground truth label instead of the manual annotation. 10% of the dataset was set apart as the test dataset. The remaining 136 3D US volumes (90%) were split into 80% training and 20% validation datasets. On default, the nnUNetv2 uses a fivefold cross-validation split. In this study, all splits were manually created on the patient level. During the preprocessing, the nnUNetv2 extracted specific properties from the dataset automatically. The 2D and 3D full resolution (3D-fullres) configurations were set up for our dataset. A new feature in nnUNetv2 is region-based training, in which the ground truth labels handle regions as overlapping areas instead of individual areas [13]. To investigate the impact of the new nnUNetv2 platform, additional data and the region-based training (RB) feature, five models were created: (1) the existing model from nnUNetv1 [8], (2) nnUNetv2 with old dataset without RB, (3) nnUNetv2 with old dataset with RB, (4) nnUNetv2 with new dataset without RB, and (5) nnUNetv2 with new dataset with RB.

### Registration

**Fig. 1:**
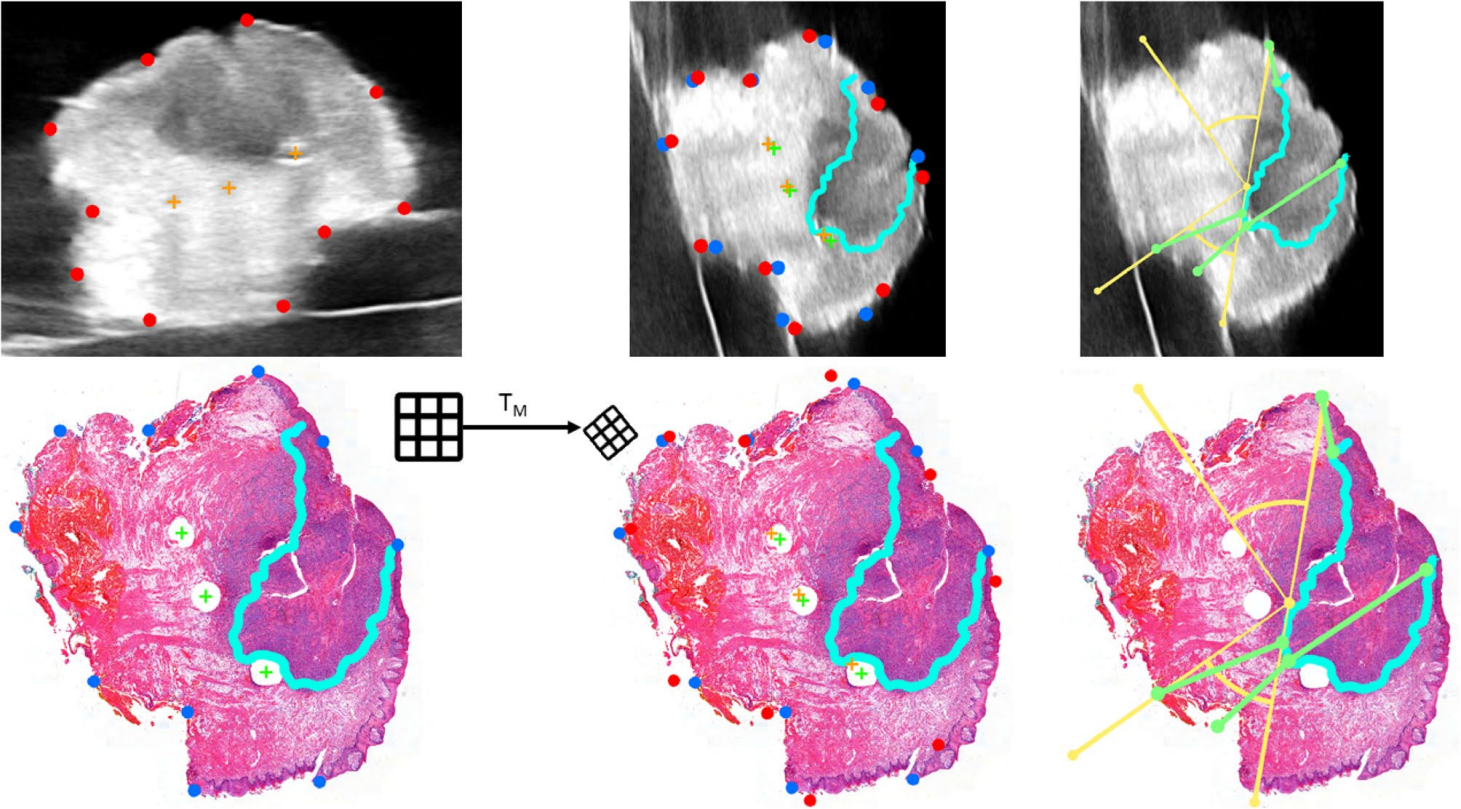
The upper row shows the ultrasound images, and the lower row shows the histopathology images from left to right, from point selection and registration to margin measurement. In the ultrasound images, the red dots represent the circumferential landmarks, and the orange crosses represent the fiducial markers. In the histopathology images, the blue dots represent the circumferential landmarks, and the green crosses the fiducial markers. The ultrasound image in the middle column shows the registration with the transformation matrix T_M_, with the histopathological tumor annotation in light blue as an overlay. In the right column, the yellow angles represent the cranial, deep, and caudal regions. The green lines represent the measurements of margins and thickness

Point lists with corresponding points were made for both the US images and the WSI. Figure 1 shows that a point list consisted of circumferential landmarks visible in both modalities and the fiducial markers. The fiducial markers were manually selected in the US image. Due to the higher resolution in the WSI, the fiducial markers could be segmented, and the center point of the marker was computed.

For each modality, four point lists were created: (1) only circumferential landmarks, (2) circumferential landmarks and the left and middle fiducial markers, (3) circumferential landmarks and the left and right fiducial markers, and (4) circumferential landmarks and the middle and right fiducial markers. As explained below, the unused fiducial markers were used as the validation marker.

A transformation matrix (T_M_) between two corresponding point lists was computed for each set of point lists. To evaluate the registration accuracy, the target registration error (TRE) between the x and y coordinates of the transformed validation marker(s) in US ($US_x$ and $US_y$) and those of the corresponding validation marker in WSI ($PA_x$ and $PA_y$) was computed, Eq. (1). For the point lists with only circumferential landmarks, all fiducial markers were validation markers, and the TRE was averaged. For the point lists, including fiducial markers, the left-out validation marker was used to compute the TRE. The T_M_ resulting in the lowest TRE was used to analyze the measurements after registration.

1 $$Target \, registration \,error = \sqrt {(US_x - PA_x )^2 + (US_y - PA_y )^2 }$$

### Tumor annotation

The US images were manually annotated by a technical physician, referred to as US_M, for the correlation and agreement analysis. A head and neck pathologist manually annotated the WSI. As explained above, the T_M_ with the lowest TRE was applied to the US image. After this registration, the HTA in the registered US image, referred to as US_Reg, was a second annotation.

### Measurements

The distance between the tumor annotation and the specimen boundary was measured in the specimen’s cranial, deep, and caudal regions. Also, the tumor thickness was measured. The measurements were performed three times: 1) in the US images with the manual tumor annotation (US_M), 2) in the registered US images with the HTA (US_Reg), and 3) in the WSI with the manual HTA.

### Statistical analysis

The Spearman’s correlation coefficient computed the correlation between the measurements in US_M and US_Reg, and WSI. Correlation between modalities was considered significant if p < 0.05. The agreement was evaluated by Bland Altman plot showing the difference between two modalities against the mean of two modalities. The correlation and agreement were computed for all measurements. Secondly, a subdivision was created by splitting the mean of the modalities into the categories < 5, 5 < 10, and > 10 mm.

### nnUNetv2: training and predicting

nnUNetv2 trained a 2D and 3D-fullres configuration for all four datasets with a maximum of 1000 epochs. nnUNetv2 automatically determined the best configuration and optional postprocessing. The predictions were performed with the best-performing configuration per dataset.

### nnUNetv2: evaluation

The test dataset of 15 held-out 3D US volumes was predicted by the nnUNetv1 and by the four best-performing configurations per dataset of nnUNetv2. The model’s performance was evaluated by the segmentation metrics, Dice similarity coefficient (DSC), 95th percentile of the Hausdorff distance (HD95), and average surface distance (ASD).

## Results

### Registration

**Fig. 2:**
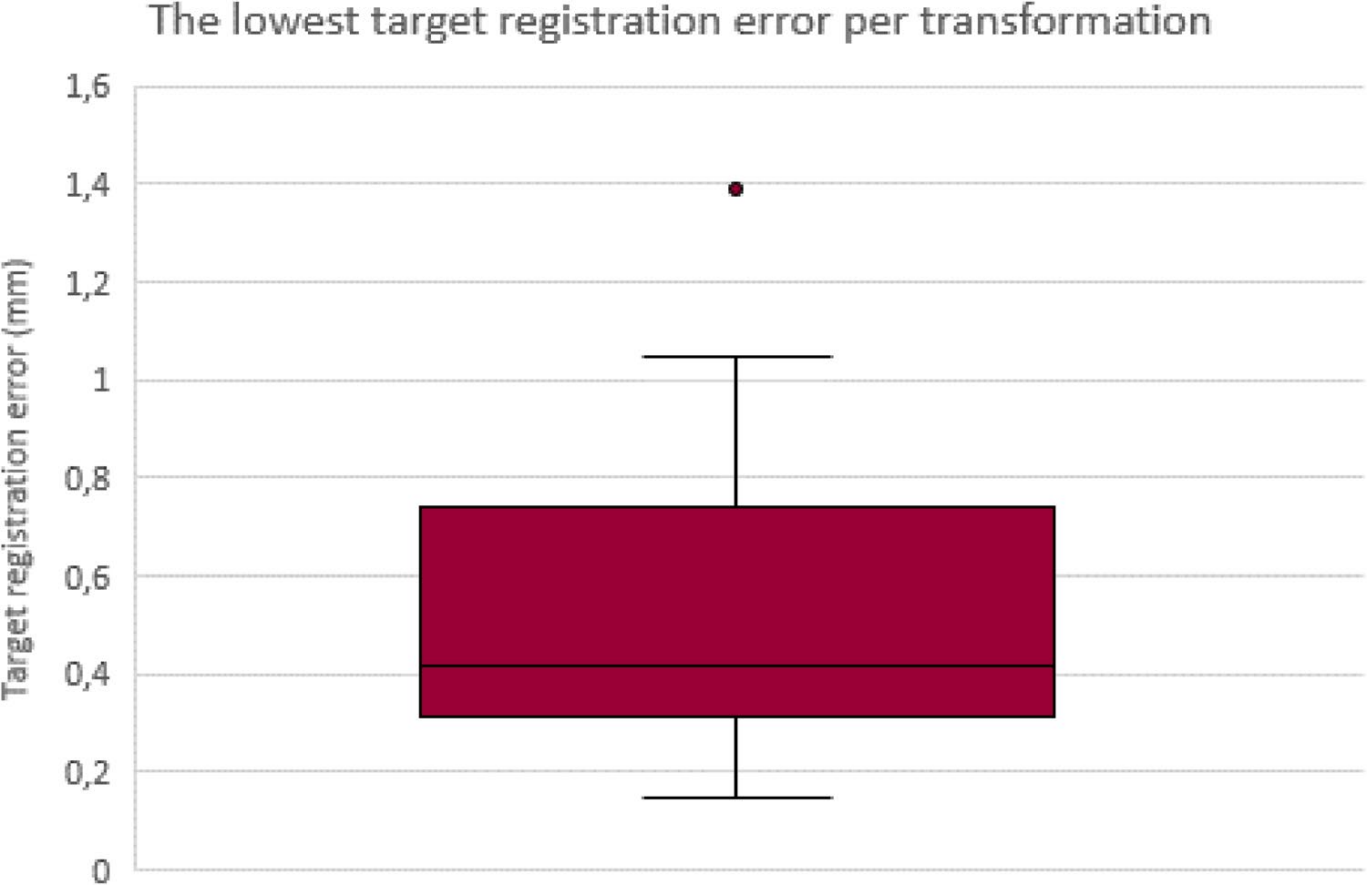
A boxplot showing the lowest target registration error per whole slide image and corresponding ultrasound image. The median target registration error is 0.42 mm

125 slides were made from the ten included patients. After excluding subdivided slides and slides without tumor, 29 WSI from eight patients were included in the study. Four point lists were created per WSI, and the corresponding US image and the transformations were computed. Figure 2 shows a boxplot of the lowest TRE per WSI obtained from the four transformations. The median TRE of the transformations from US to WSI was 0.42 mm.

Table 1 shows Spearman’s correlation coefficient between margin and tumor thickness measured based on the US_M and US_Reg, compared to the WSI. The correlation between all measurements for both US_M and US_Reg, and histopathology was significant, as well as for the categories < 5 and 5 < 10 mm. The category > 10 mm of mean measurements of the two modalities did not show a significant correlation between the US_M and US_Reg, and histopathology. Comparing US_M with US_Reg, the latter showed a higher correlation for all measurements and the different categories.

**Table 1:** The Spearman’s correlation coefficient computed between the margin and tumor thickness measurements in ultrasound with manual and registered annotation, and histopathology

Measurements	Spearman’s correlation coefficient between 3D ultrasound and histopathology	
	Manual annotation (US_M)	Registered annotation (US_Reg)
All	*0.719 (p* < *0.001)*	*0.924 (p* < *0.001)*
< 5 mm	*0.554 (p* = *0.014)*	*0.792 (p* < *0.001)*
5 < 10 mm	*0.589 (p* < *0.001)*	*0.727 (p* < *0.001)*
> 10 mm	− 0.172 (p = 0.468)	0.410 (p < 0.34)

The correlation was calculated for all measurements and split into the categories < 5, 5 < 10, and > 10 mm of the mean of the two modalities. The correlations in italics are statistically significant

### Agreement

**Fig. 3:**
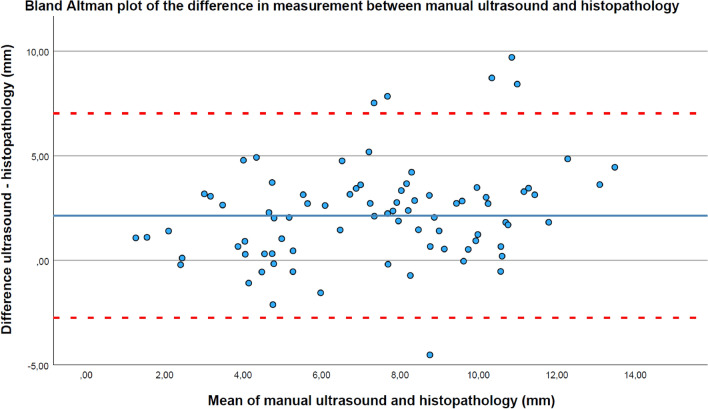
A Bland Altman plot of the difference in measurement between manually annotated tumors in US and histopathology. The blue horizontal line is the mean difference between ultrasound and histopathology. The two red dashed lines are the lower and upper limits of agreement of the 95% confidence interval. The mean difference and 95% confidence interval is 2.14 mm (− 2.75; 7.03)

**Fig. 4:**
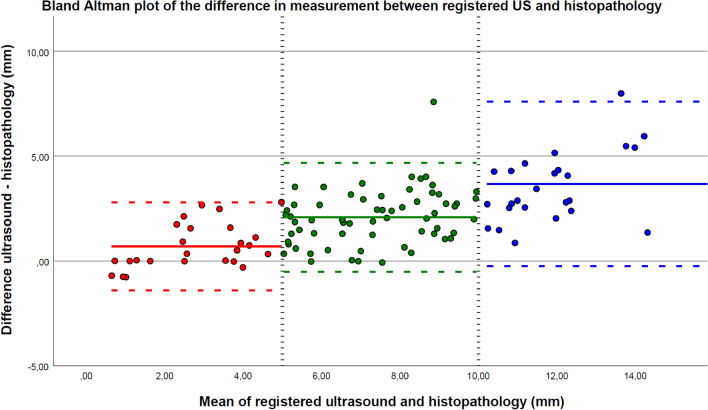
A Bland Altman plot of the difference in measurement between the registered tumor annotation in US and histopathology. The vertical black dashed lines at 5 and 10 mm (x-axis) divided the data into the categories < 5 (red), 5 < 10 (green), and > 10 mm (blue) mean measurements of histopathology and registered US. The horizontal line indicates the mean difference between ultrasound and histopathology for each category. The dotted lines indicate the upper and lower limits of agreement of the 95% confidence interval. The mean difference and 95% confidence interval for the category < 5 is 0.69 mm (− 1.40; 2.78), 5 < 10 is 2.08 mm (− 0.52; 4.68), and for > 10 is 3.68 mm (− 0.24; 7.61). *US* ultrasound

Figure 3 shows the agreement in measurements between US_M and histopathology. For the agreement between US_Reg and histopathology, the Bland Altman plot is shown in Fig. 4. The agreement for all measurements and categories between US_M and US_Reg, and histopathology is shown in Table 2. The mean difference between US_M and histopathology, and US_Reg and histopathology was almost similar, except for the category < 5 mm. In this category, the mean difference between the modalities was reduced from 1.29 to 0.69 mm. For all measurements and all categories, the 95%CI became smaller for the US_Reg.

**Table 2:** The agreement between the margin and tumor thickness measurements based on the manual and registered annotation and histopathology

Measurements	Agreement between 3D ultrasound and histopathology	
	Mean difference of manual annotation (US_M) in mm (95%CI)	Mean difference of registered annotation (US_Reg) in mm (95%CI)
All	2.14 (− 2.75; 7.03)	2.16 (− 1.31; 5.63)
< 5 mm	1.29 (− 2.24; 4.82)	0.69 (− 1.40; 2.78)
5 < 10 mm	2.02 (− 2.75; 6.78)	2.08 (− 0.52; 4.68)
> 10 mm	3.59 (− 2.16; 9.34)	3.68 (− 0.24; 7.61)

*CI* confidence interval

### nnUNetv2

**Fig. 5:**
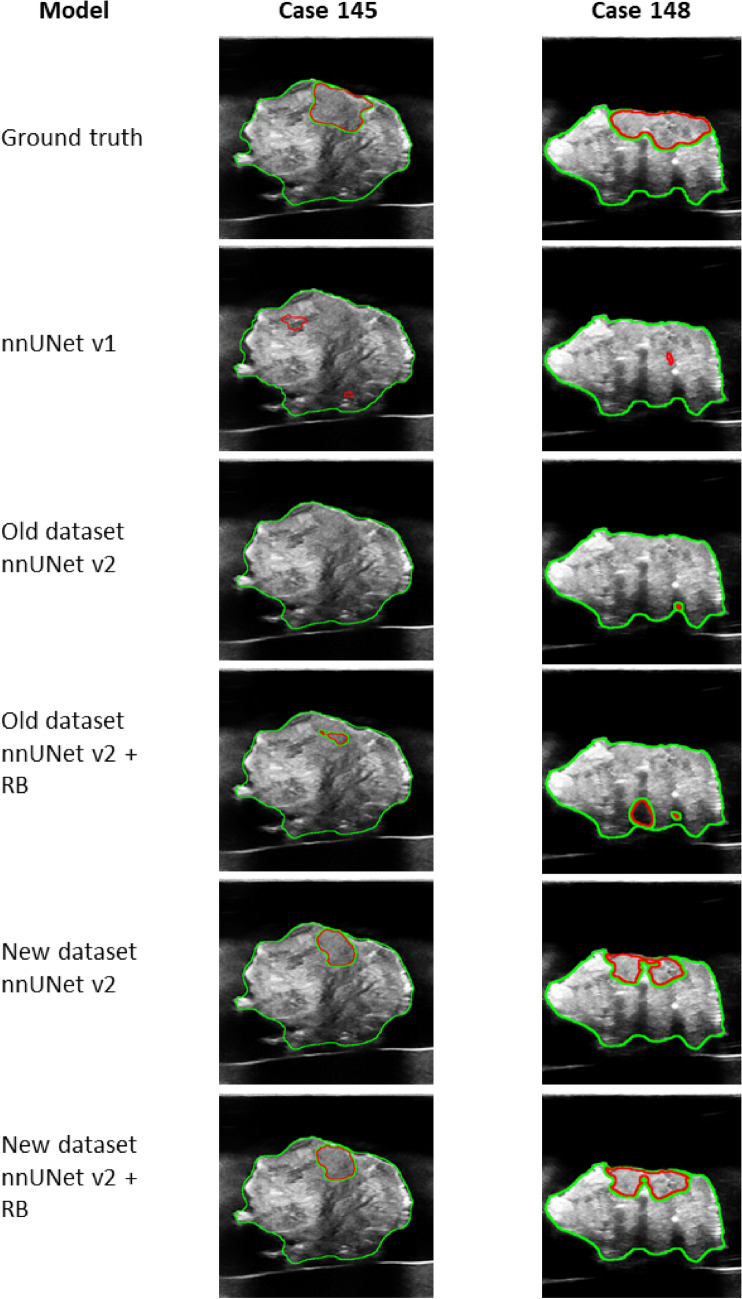
The ground truth annotation and the predictions of the different models on two cases from the held-out test set. The predictions shown are based on the ensembled models, which include postprocessing steps. The specimen is outlined in green and the tumor in red. *RB* region-based training

The five models predicted the 15 3D US volumes from the test set. The pixel spacing of the volumes was 0.076 × 0.076, with a slice spacing of 0.5 mm. Figure 5 shows the predictions of the different models of two cases, including the corresponding ground truth annotation. Both cases showed a clear difference between the models trained with the old and new datasets. The results of all models for the specimen region, in Table 3, were within a close range: DSC of 0.952–0.971, ASD of 0.21–0.29 mm, and the HD95 of 1.17–1.66 mm. Table 4 shows larger differences in the models’ performance for the tumor region. The models trained with nnUNetv2 with the extended dataset outperformed the models of both nnUNet v1 and v2, which were trained with the old dataset. The DSC improved from 0.455 to 0.621, the ASD from 1.69 to 1.05 mm, and the HD95 from 5.99 to 3.70 mm. The influence of the nnUNetv2 platform or the region-based training was not noticeable in the performance results.

**Table 3:** The performance metrics of the different models on the specimen region

Specimen				
Model	All or ensemble	Dice Similarity coefficient	Average surface distance (mm)	95th percentile of Hausdorff distance (mm)
Old dataset v1	All	**0.971**	**0.21**	1.44
	Ensembled	0.956	0.29	1.64
Old dataset v2	All	0.953	0.27	1.66
	Ensembled	0.957	0.23	1.50
Old dataset v2 + RB	All	0.952	0.26	1.63
	Ensembled	0.957	0.24	1.50
New dataset v2	All	0.960	0.25	1.20
	Ensembled	0.961	0.24	1.19
New datasetv2 + RB	All	0.960	0.26	**1.17**
	Ensembled	0.961	0.23	**1.17**

The values represent the averages based on the 15 3D US volumes in the test set. The best values per metric are in bold

*RB* region-based training

**Table 4:** The performance metrics of the different models on the tumor region

Tumor				
Model	All or ensemble	Dice Similarity coefficient	Average surface distance (mm)	95th percentile of Hausdorff distance (mm)
Old dataset v1	All	0.455	2.39	6.64
	Ensembled	0.364	2.70	7.20
Old dataset v2	All	0.364	2.36	7.34
	Ensembled	0.397	1.90	6.78
Old dataset v2 + RB	All	0.431	1.69	5.99
	Ensembled	0.428	2.44	6.29
New dataset v2	All	**0.621**	1.15	**3.70**
	Ensembled	0.586	**1.05**	3.94
New datasetv2 + RB	All	0.610	1.21	3.82
	Ensembled	0.588	1.08	3.95

The values represent the averages based on the 15 3D US volumes in the test set. The best values per metric are in bold

*RB* region-based training

## Discussion

The goal of this study was to create ground truth annotation of US images using HTA. The objectives were (1) to investigate the registration accuracy of 3D US to postoperative histopathology slides, (2) to assess the agreement and correlation between the resection margin and tumor thickness in histopathology and US with registered annotation, and (3) to train a new version of the deep learning model for automatic segmentation of ex-vivo tongue carcinoma in 3D US volumes. The primary outcomes were that the median TRE from 3D US to histopathology was 0.42 mm, registration of the HTA in 3D US improved the agreement by 2.84 mm narrower confidence interval boundaries and improved the correlation to very strong (r = 0.924). The nnUNetv2 model trained on a large dataset showed an improved segmentation performance for the tumor region based on DSC (0.621), ASD (1.15), and HD95 (3.70). These improvements indicated that automatic margin assessment is progressing toward clinical implementation.

The novel aspect of this study was the registration of 3D US to histopathology images of resected tongue squamous cell carcinomas. Based on fiducial markers and visible landmarks, we obtained a median TRE of 0.42 mm between the ex vivo 3D US and histopathology slices. This is a small error compared to studies about the prostate, a field where multimodal registration is more advanced [4–6, 14]. Li et al. compared different registration methods and reported a root mean squared distance between the corresponding histopathological landmarks and the in vivo prostate MRI from 2.96 to 3.97 mm [5]. In a review, MRI or US were registered to the histopathology and validated by corresponding landmarks, surface overlap, or visual inspection. For US, the TRE of the manual landmarks ranged from 2.1 to 3.8 mm. Shao et al. reported a Dice overlap score of 0.5 as a promising accuracy of the alignment of cancer tissue [6]. These overlap scores cannot be compared to the TRE from our study. Comparing the Dice score of 0.61 for the tumor region in our dataset of 3D US volume, our nnUNet segmentation models performed less than other head and neck reported outcomes such as the oropharyngeal cancer segmentation in CT and MRI with nnUNet by Choi et al. [15]. To our knowledge, segmentation studies similar to the current study have not been reported.

The strength of this study was the multimodal image registration approach to obtain US ground truth labels with histopathological accurateness. We explained how the registration was performed and quantitatively evaluated, which resulted in an improved agreement and correlation between the measurements in 3D US and histopathology. Highlighting the margin category < 5 mm as this is clinically relevant, the agreement was 0.69 mm with a correlation of 0.792. This confirmed the use of registered HTA to annotate the ground truth labels. Another strong aspect is the training of a new deep learning model. The most recent version of the nnUNetv2 platform, combined with an extended dataset, 29 slices of registered HTA ground truth labels, and region-based training, provided higher quantitative evaluation scores of the model’s performance. The impact of the version 2 platform and region-based training was not directly noticeable. Furthermore, 29 slices of registered HTA ground truth labels were negligible, as the dataset contained 151 3D US volumes of around 140 slices each. This substantiates the need to continuously acquire data to improve the model’s segmentation performance.

Some aspects of the study might have introduced weaknesses. Registration between 3D US and histopathology is based on corresponding landmarks, which were manually selected except for the fiducial markers in the WSI. The US images had a pixel size of 0.076 mm; thus, selecting a couple of pixels next to the actual representing pixels of the fiducial marker could provide results with a deviation of around 0.5 mm. Automatic registration algorithms, such as Synthmorph [16], could reduce the effect of manual selection. We preferred the possibility of adjusting the registration process as the fresh resected tissue could be deformed or damaged, so these regions in the image should be ignored during the registration.

The TRE after registration showed a median of 0.42 mm. One outlier of 1.39 mm was observed, which was the final slice in the specimen with only one fiducial marker visible. Therefore, the registration was performed based on the visual landmarks, as this fiducial marker had to be used as a validation marker. This indicated that registration with only visual landmarks at the circumference could result in higher TRE. Removing this outlier from the analysis resulted in a median TRE of 0.41 mm, indicating the small impact of this outlier.

The agreement between the measurements in histopathology and manually annotated US, and registered HTA in US resulted in similar mean differences. The 95% CI width was reduced from 9.78 to 6.94 mm by the registered HTA in US. This was likely due to the fact that in the US images, small irregular growth patterns were not missed, and tumor infiltration processes were not annotated as tumor. These improvements resulted in a very strong statistically significant correlation of 0.924 (p < 0.001). Subdivided into three categories < 5, 5 < 10, and > 10 mm, a lower mean difference of 0.69 mm (95% CI − 1.40; 2.78) was observed in the < 5 mm category with a smaller 95% CI. This means that measurements < 5 mm based on the registered HTA in US strongly correlated and agreed with histopathology. This category is clinically important as a close margin indicates adjuvant therapy, such as second surgery or (chemo)radiation [17]. For the categories 5 < 10 and > 10 mm, the mean differences were similar with smaller 95%CI for the registered HTA in US.

Worth highlighting is the fact that larger measurements were associated with larger mean differences with wider 95% CI and less strong correlations, as visualized in Fig. 4. This could be the consequence of tissue shrinkage between the 3D US acquisition and the digitalization of fixated and paraffin-embedded histopathology slices [18, 19]. Shrinkage occurs relative to the entire resected specimen. Measuring distance over a shorter distance, such as in the category < 5 mm, the relative shrinkage will be equal to measurements over a larger distance. However, in absolute numbers, this shrinkage resulted in submillimeter deviation. Overall, the improved agreement and correlation between registered HTA in US and histopathology substantiated that multimodal image registration was preferred over manual annotation of 3D US volumes to obtain high-accuracy ground truth labels for future deep learning models.

The segmentation models performed similarly for the specimen region, although there were differences in dataset size and region-based training. For the tumor region, the models’ performances were mostly divergent by the dataset size. Extending the dataset resulted in an almost 0.2 increase in DSC, a decrease of around 0.5 mm in the ASD, and around 30% smaller HD95. Besides providing the model with more data, we believe the data quality has improved over the years. The first 3D US volumes of the old dataset were acquired by electromagnetically tracking the US transducer as described in a previous study [2]. The motorized approach for 3D US acquisition provided more stable and less variable 3D US acquisitions [7]. The new dataset was an extension with only the 3D US volume acquired by the latter approach.

The new dataset also contained the registered HTA in the US images as ground truth labels. However, the effect of these high-quality ground truth labels on the final model’s performance was negligible. Only 29 images received these ground truth labels. These high-quality ground truth labels were only 0.15% of the total dataset. Whereas, the training and validation dataset contained 136 3D US volumes, each comprising around 140 US images. Adding more data improves the model’s performance [20] as long as the acquisition and annotation quality can be assured. At some point, a slight improvement requires excessive data, and whether that improvement is still clinically relevant could be raised.

The study’s findings were clinically relevant as fast and automatic segmentation of the 3D US volumes of the resected tongue specimen could help assess the resection margins intra-operatively. The multimodal image registration from 3D US to histopathology demonstrated an approach to improve the quality of the ground truth labels of potentially the nnUNet model’s performance. Additionally, the extended dataset increased the segmentation performance of the nnUNet, which encouraged the prospective acquisition of more 3D US volumes. The next step in this research would be to study the impact of the intra-operative assessment, including the nnUNet model, on the detection of close margins in resected tongue specimens.

In conclusion, US images can be registered to WSI with submillimeter accuracy. The multimodal image registration enabled the overlay of registered HTA in US images, which resulted in an agreement up to 0.69 mm and a very strong correlation (r = 0.924, p < 0.001) with histopathology, especially in the < 5 mm resection margin category. These numbers substantiate the use of multimodal image registration to obtain high-quality ground truth labels for deep learning models. Besides improving the data quality, extending the nnUNet model’s dataset resulted in improved performance towards clinical applicability.

## Data Availability

Data is available upon request via the corresponding author.
